# Screening and management of bladder and bowel dysfunction in general pediatric outpatient clinic: a prospective observational study

**DOI:** 10.1186/s12887-022-03360-9

**Published:** 2022-05-17

**Authors:** Achra Sumboonnanonda, Punnarat Sawangsuk, Patharawan Sungkabuth, Janpen Muangsampao, Walid A. Farhat, Nuntawan Piyaphanee

**Affiliations:** 1 Division of Nephrology, Department of Pediatrics, Faculty of Medicine Siriraj Hospital, Mahidol University, 2 Wanglang Road, Bangkoknoi, Bangkok, 10700 Thailand; 2 Pediatric Nursing Division, Department of Nursing, Faculty of Medicine Siriraj Hospital, Mahidol University, Bangkok, Thailand; 3 Department of Urology, University of Wisconsin, Madison, WI USA

**Keywords:** Screening, Management, Bladder and bowel dysfunction, General pediatric outpatient clinic, Siriraj Hospital, Thailand, DVSS

## Abstract

**Background:**

Bladder and bowel dysfunction (BBD) is a common disorder in children that is often associated with psychosocial and behavioral problems. Data specific to BBD in Asian children are comparatively scarce. Accordingly, this study aimed to investigate the prevalence of BBD and the response to standard urotherapy among Thai children attending the general pediatric outpatient clinic of Siriraj Hospital – Thailand’s largest national tertiary referral center.

**Methods:**

Children aged 4–12 years were recruited to complete the Dysfunctional Voiding Symptom Score (DVSS) questionnaire to screen for BBD during 2018 to 2020. Standard urotherapy, which consists of education and behavior management, was prescribed to those with a DVSS score that suggests the presence of BBD. Enrolled children and their caregivers were followed-up at 3 and 6 months. The Strengths and Difficulties Questionnaire (SDQ) was completed at baseline. DVSS scores at baseline, and at 3 months and 6 months after standard urotherapy were compared using repeated measures analysis of variance (ANOVA).

**Results:**

A total of 1,042 children completed the DVSS during the study period, and 90 (8.6%) were deemed to have BBD. The mean age of BBD children was 6.9 ± 2.2 years, and the female to male ratio was 2.9:1. The most common symptoms were defecation frequency (80.0%), difficult defecation (80.0%), curtsying (74.4%), urgency (68.9%), infrequent voiding (43.3%), and daytime incontinence (32.2%). Forty-one BBD children completed the SDQ, and 32.5% had scores suggestive of hyperactivity problems. Among the 24 BBD patients who were followed-up after 3 and 6 months of standard urotherapy, there was a significant improvement in DVSS results (9.5 ± 3.1 at baseline *vs.* 6.9 ± 3.4 at 3 months *vs.* 4.4 ± 3.9 at 6 months; *p* < 0.01). Nine of 12 patients with urinary incontinence showed complete response at 6 months. The overall Bristol stool score significantly improved from 2.6 ± 0.7 at baseline to 3.2 ± 1.0 at 6 months (*p* = 0.03).

**Conclusions:**

BBD is a prevalent condition among Thai children that is often associated with emotional and behavioral problems. Standard urotherapy prescribed in a general pediatric outpatient clinic setting yielded favorable outcomes in Thai children with BBD.

## Background

Bladder and bowel dysfunction (BBD) comprises lower urinary tract dysfunction (LUTD) and bowel dysfunction. LUTD can present with symptoms that primarily include incontinence, abnormal urinary frequency, urgency, hesitancy, straining, weak stream, intermittency, and dysuria. Bowel dysfunction commonly manifests as primary constipation and/or encopresis [1, 2]. BBD is common in children worldwide, and is associated with psychosocial and behavioral problems [3–6]. The prevalence of BBD in school-age children was reported to range from 9.1–21.8% [3, 4, 7]; however, data specific to the prevalence of BBD in Asian children, including Thai children, remain scarce.

The Dysfunctional Voiding Symptom Score (DVSS) questionnaire was found to be a useful tool for screening and diagnosing BBD, as well as for evaluating the outcome of treatment [8–10]. The Strengths and Difficulties Questionnaire (SDQ) has been widely used to evaluate emotional and behavioral problems in children aged 2–17 years [11–13]. Standard urotherapy, which is given to both the patient and the caregiver, is non-pharmacological and non-surgical management that consists of education and behavior management using a bladder and bowel diary, and encouragement via regular follow-up [1, 14]. Education given to both the child and the caregiver includes voiding function and demystification, proper and regular bladder and bowel habits, and balanced fluid intake and diet.

Recognition and non-invasive management of BBD in a general outpatient clinic setting by a general practitioner or a non-subspecialist pediatrician may be helpful for avoiding further complications, such as urinary tract infection (UTI) and psychological complications. To improve our understanding of BBD in Asian pediatric population, this study set forth to examine the prevalence of BBD and the response to standard urotherapy among Thai children attending the general pediatric outpatient clinic of Siriraj Hospital, which is a 2,300-bed university-based national referral hospital that is located in Bangkok, Thailand.

## Methods

The prospective observational study enrolled pediatric patients aged 4–12 years who attended the general pediatric outpatient clinic of Siriraj Hospital and who agreed to take the Thai version of the DVSS questionnaire. The questionnaire was completed by the patient and/or a parent/caregiver/legal guardian. Children attending our clinic for acute illness, follow-up visit after previous illness, or for health supervision were recruited. Demographic and clinical data, including age, gender, parental marital status, number of siblings, family income, underlying disease, and history of UTI, were recorded. Verbal assent and written informed consent to participate in this study was obtained from study children and their parent/caregiver/legal guardian, respectively. This study was approved by the Siriraj Institutional Review Board and was performed in accordance with the Declaration of Helsinki.

The Thai version of the DVSS questionnaire consists of 10 items, including 7 items related to voiding, 2 items related to defecation, and 1 item related to stressful events. The score for each item ranges from 0 to 3 corresponding with 4 levels of frequency over the previous month (0 = almost never, 1 = less than half the time, 2 = about half the time, and 4 = almost all the time). DVSS cutoff values of 9 in boys and 6 in girls were reported to diagnose BBD [8, 9].

Standard urotherapy regimen, including frequent voiding during the day (4–7 voids/day), appropriate fluid intake (minimum of 1–2.5 L per day), proper voiding posture, avoidance of holding maneuver, avoidance of caffeine containing beverage, high fiber diet encouragement, and regular bowel habit [1], was prescribed to children with BBD. The BBD children who agreed to participate in further study that included treatment via standard urotherapy completed the Thai version of Strengths and Difficulties Questionnaire (SDQ) [12], and were followed-up by nurses trained in BBD management. Timed voiding and double voiding were advised if indicated, and laxative and/or enema were prescribed by a pediatrician as indicated. During the first two weeks of treatment, participants were asked to record their 24-h bladder diary for two days and record their bowel diary including Bristol stool scale for 7 days. Bladder and bowel diary were recorded again at 6 months. An information booklet that included a summary of the goal of urotherapy was given as a take home reference and frequent reminder to study participants. Patients and/or their parent/caregiver/legal guardian completed the DVSS questionnaire at both the 3- and 6-month follow-ups.

Urinary incontinence was recorded in the bladder diary, and treatment outcome was defined as no-response (< 50% reduction), partial response (50–99% reduction) or complete response (100% reduction) according to International Children’s Continence Society definition [1].

### Statistical analysis

Patient demographic and clinical data were summarized using descriptive statistics. Comparisons of categorical data were performed using Fisher’s exact test or chi-square test depending on the size of the sample, and the results of those comparisons are given as frequency and percentage. Comparisons of normally distributed continuous data were performed using independent samples *t*-test, and those results are shown as mean ± standard deviation (SD). Baseline characteristics and total DVSS score of those who completed 6 months of standard urotherapy were compared with those of patients who did not complete 6 months of standard urotherapy to identify any potential biases. DVSS results were compared among baseline, and 3 and 6 months after standard urotherapy using repeated measures analysis of variance (ANOVA). All statistical analyses were performed using PASW Statistics version 18.0 (SPSS, Inc., Chicago, IL, USA), A *p*-value less than 0.05 was considered statistically significant for all tests.

## Results

A total of 1,042 children completed the DVSS questionnaire at our center’s general pediatric outpatient clinic during the 2018 to 2020 study period. The female to male ratio was 2.9:1. Using the established DVSS cutoff values, 90 children (8.6%) were found to harbor BBD. Except for the relative proportion of female gender, which was found to be significantly higher in the BBD group than in the non-BBD group, there were no other significant differences in the evaluated demographic and clinical characteristics of patients between the BBD and non-BBD groups (Table 1).

**Table 1:** Demographic and clinical data of children compared between those with and without BBD

Demographic and clinical data	BBD (*n* = 90)	No BBD (*n* = 952)	*p*-value
Age (years), mean ± SD	6.9 ± 2.2	7.1 ± 2.3	0.293
Age ≤ 5 years, n (%)	21 (23.3%)	218 (22.9%)	0.931
Age 5–10 years, n (%)	56 (62.2%)	591 (62.1%)	0.985
Age > 10 years, n (%)	13 (14.4%)	143 (15.0%)	0.879
Female gender, n (%)	67 (74.4%)	412 (43.3%)	**< *0.001***
Parents living together, n (%)	71 (78.9%)	714 (75.0%)	0.412
Number of siblings, mean ± SD	1.89 ± 0.73	1.79 ± 0.75	0.213
Monthly family income (THB/month), n (%)	19 (21.1%)	149 (15.7%)	0.184
< 10,000	36 (40.0%)	341 (35.8%)	0.428
10,000–25,000	26 (28.9%)	313 (32.9%)	0.439
25,001–50,000	9 (10.0%)	149 (15.7%)	0.150
> 50,000	32 (35.6%)	284 (29.8%)	0.280
Underlying disease, n (%)			
Any underlying disease	32 (35.6%)	284 (29.8%)	0.280
Allergic disorder	22 (24.4%)	203 (21.3%)	
Neuropsychiatric disorder	6 (6.7%)	18 (1.9%)	
History of UTI, n (%)	2 (2.2%)	12 (1.3%)	0.344

*Abbreviations: BBD* bladder and bowel dysfunction, *SD* standard deviation, *THB* Thai baht, *UTI* urinary tract infection

A *p*-value < 0.05 indicates statistical significance

From the DVSS questionnaire, the most common symptoms of BBD compared to the prevalence reported by non-BBD patients were low defecation frequency (80.0% *vs.* 43.2%), difficult defecation (80.0% *vs.* 38.9%), curtsying (74.4% *vs.* 32.1%), urgency (68.9% *vs.* 28.2%), infrequent voiding (43.3% *vs.* 16.4%), and daytime incontinence (32.2% *vs.* 5.5%) – all respectively. The mean score for each of the 10 DVSS questions was significantly higher in the BBD group than in the non-BBD group. Accordingly, the overall DVSS result was significantly higher in the BBD group than in the non-BBD group (8.9 ± 2.9 *vs.* 2.5 ± 2.0, respectively; *p* < 0.01) (Table 2).

**Table 2:** Mean (± standard deviation) result for each DVSS question and for the total DVSS result among all study children, and compared between those with and without BBD

DVSS questions	Total (*N* = 1,042)	BBD (*n* = 90)	No BBD (*n* = 952)	*p*-value
Q1 (daytime incontinence)	0.1 ± 0.4	0.5 ± 0.9	0.1 ± 0.3	**< *0.01***
Q2 (wetting amount)	0.1 ± 0.4	0.5 ± 0.9	0.1 ± 0.3	**< *0.01***
Q3 (low defecation frequency)	0.8 ± 0.96	1.6 ± 1.1	0.7 ± 0.9	**< *0.01***
Q4 (difficult defecation)	0.6 ± 0.8	1.5 ± 1.1	0.5 ± 0.8	**< *0.01***
Q5 (infrequent voiding)	0.3 ± 0.7	0.9 ± 1.2	0.2 ± 0.6	**< *0.01***
Q6 (curtsying)	0.5 ± 0.8	1.6 ± 1.2	0.4 ± 0.6	**< *0.01***
Q7 (urgency)	0.4 ± 0.7	1.3 ± 1.1	0.3 ± 0.6	**< *0.01***
Q8 (push to void)	0.1 ± 0.3	0.2 ± 0.6	0.1 ± 0.3	***0.01***
Q9 (dysuria)	0.1 ± 0.3	0.3 ± 0.6	0.0 ± 0.2	**< *0.01***
Q10 (stressful events)	0.1 ± 0.5	0.5 ± 1.1	0.1 ± 0.4	***0.01***
Total DVSS (0–30)	3.0 ± 2.8	8.9 ± 2.9	2.5 ± 2.0	**< *0.01***

*Abbreviations: DVSS* Dysfunctional voiding symptom score, *BBD* Bladder and bowel dysfunction, *Q* Question

A *p*-value < 0.05 indicates statistical significance (comparison between BBD and non-BBD patients)

A total of 41 (45.6%) BBD patients initially agreed to participate in this prospective study. A comparison of those 41 BBD patients with the 49 BBD patients that did not agree to or did not complete 6 months of standard urotherapy revealed no significant differences in mean age (*p* = 0.30), gender (*p* = 0.63), parents living together (*p* = 0.74), number of siblings (*p* = 0.65), or family income (*p* = 0.30). In the present study, we found 17.5% of BBD children to be at risk for emotional and behavioral problems (17.5%), and that 20.0% of BBD children had emotional and behavioral problems according to overall SDQ scale scoring. The most common domains were the emotional (15, 20.0%), conduct (12.5, 7.5%), hyperactive (5.0, 32.5%), and peer (20.0, 7.5%) domains, and 22.5% of children had low pro-social scores.

A total of 32 (78.0%) patients completed 3 months of standard urotherapy and came to the 3-month follow-up visit. The mean total DVSS result of those 32 participants improved significantly from 9.4 ± 2.9 at baseline to 6.6 ± 3.5 at 3 months (*p* < 0.01). Four of 10 individual symptoms also improved significantly, including low defecation frequency (*p* = 0.02), infrequent voiding (*p* = 0.01), curtsying (*p* = 0.02), and stressful events (*p* = 0.04). Of these 32 patients, laxative treatment was prescribed in 5 (15.6%) patients with 1 patient also needed enema.

A total of 24 (58.5%) patients completed 6 months of standard urotherapy and attended the 6-month follow-up visit. The baseline characteristics and mean total DVSS result of those who completed 6 months of standard urotherapy (*n* = 24) were not significantly different from those of BBD children who did not complete 6 months of standard urotherapy (*n* = 66) (Table 3). The mean total DVSS of those 24 participants decreased significantly from 9.5 ± 3.1 at baseline to 6.9 ± 3.4 at 3 months, and then decreased further to 4.4 ± 3.9 (*p* < 0.01) at 6 months. The mean overall DVSS result and the mean results of all 10 individual symptoms all showed significant improvement when compared among baseline and 3 and 6 months after standard urotherapy (Table 4). Three (12.5%) patients were treated with laxative, and then 2 patients could be discontinued the treatment.

**Table 3:** Baseline characteristics of the 90 enrolled children with BBD compared between those who did and did not complete 6 months of treatment

Characteristics	All enrolled children with BBD (*n* = 90)		*p*
	**BBD who completed 6 months of treatment** **(*n* = 24)**	**BBD who did not complete 6 months of treatment** **(*n* = 66)**	
Age (years), mean ± SD	7.0 ± 2.2	6.8 ± 2.3	0.71
≤ 5 years, n (%)	5 (20.8%)	16 (24.2%)	0.94
5–10 years, n (%)	15 (62.5%)	41 (62.1%)	0.97
> 10 years, n (%)	4 (16.7%)	9 (13.6%)	0.71
Female gender, n (%)	17 (70.8%)	50 (75.8%)	0.63
Parents living together, n (%)	20 (83.3%)	50 (75.8%)	0.76
Number of siblings, mean ± SD	2.5 ± 0.6	2.2 ± 0.5	0.11
Family monthly income (THB/month), n (%)			
< 10,000	3 (12.5%)	16 (24.2%)	0.37
10,000–25,000	12 (50.0%)	24 (36.4%)	0.25
25,001–50,000	8 (33.3%)	18 (27.3%)	0.58
> 50,000	1 (4.2%)	8 (12.1%)	0.27
Any underlying disease, n (%)	8 (33.3%)	24 (36.4%)	0.79
History of UTI, n (%)	1 (4.2%)	1 (1.5%)	1.00
Total DVSS result, mean ± SD	9.5 ± 3.1	8.6 ± 2.8	0.21

*Abbreviations: BBD* Bladder and bowel dysfunction, *SD* standard deviation, *THB* Thai baht, *UTI* urinary tract infection

A *p*-value < 0.05 indicates statistical significance

**Table 4:** Mean (± standard deviation) result for each DVSS question and for the total DVSS at each study time point among the 24 children with BBD who completed 6 months of treatment

DVSS questions	n (%)	Score			*p*-value
		**Baseline**	**At 3 months**	**At 6 months**	
Q1 (daytime incontinence)	8 (33.3%)	1.6 ± 0.9	0.6 ± 0.9	0.3 ± 0.7	***0.01***
Q2 (wetting amount)	6 (25.0%)	2.0 ± 0.9	1.0 ± 1.3	0.2 ± 0.4	**< *0.01***
Q3 (low defecation frequency)	20 (83.3%)	2.4 ± 0.8	1.6 ± 1.2	1.2 ± 1.1	**< *0.01***
Q4 (difficult defecation)	18 (75.0%)	2.0 ± 0.8	1.4 ± 1.0	1.1 ± 1.0	***0.02***
Q5 (infrequent voiding)	13 (54.2%)	2.0 ± 0.9	1.0 ± 1.1	0.5 ± 1.1	**< *0.01***
Q6 (curtsying)	17 (70.8%)	2.1 ± 1.0	1.5 ± 1.1	0.8 ± 1.1	**< *0.01***
Q7 (urgency)	13 (54.2%)	2.1 ± 0.8	1.3 ± 1.3	0.7 ± 1.0	**< *0.01***
Q8 (push to void)	2 (8.3%)	2.0 ± 1.4	0.5 ± 0.7	0.0 ± 0.0	0.19
Q9 (dysuria)	8 (33.3%)	1.5 ± 0.8	0.9 ± 0.8	0.4 ± 0.5	***0.03***
Q10 (stressful events)	5 (20.8%)	3.0 ± 0.0	0.6 ± 1.3	0.6 ± 1.3	***0.02***
Total (0–30)	24 (100%)	9.5 ± 3.1	6.9 ± 3.4	4.4 ± 3.9	**< *0.01***

*Abbreviations: DVSS* Dysfunctional voiding symptom score, *BBD* Bladder and bowel dysfunction, *Q* Question

A *p*-value < 0.05 indicates statistical significance among the 3 time points (repeated measures analysis of variance)

**Fig. 1:**
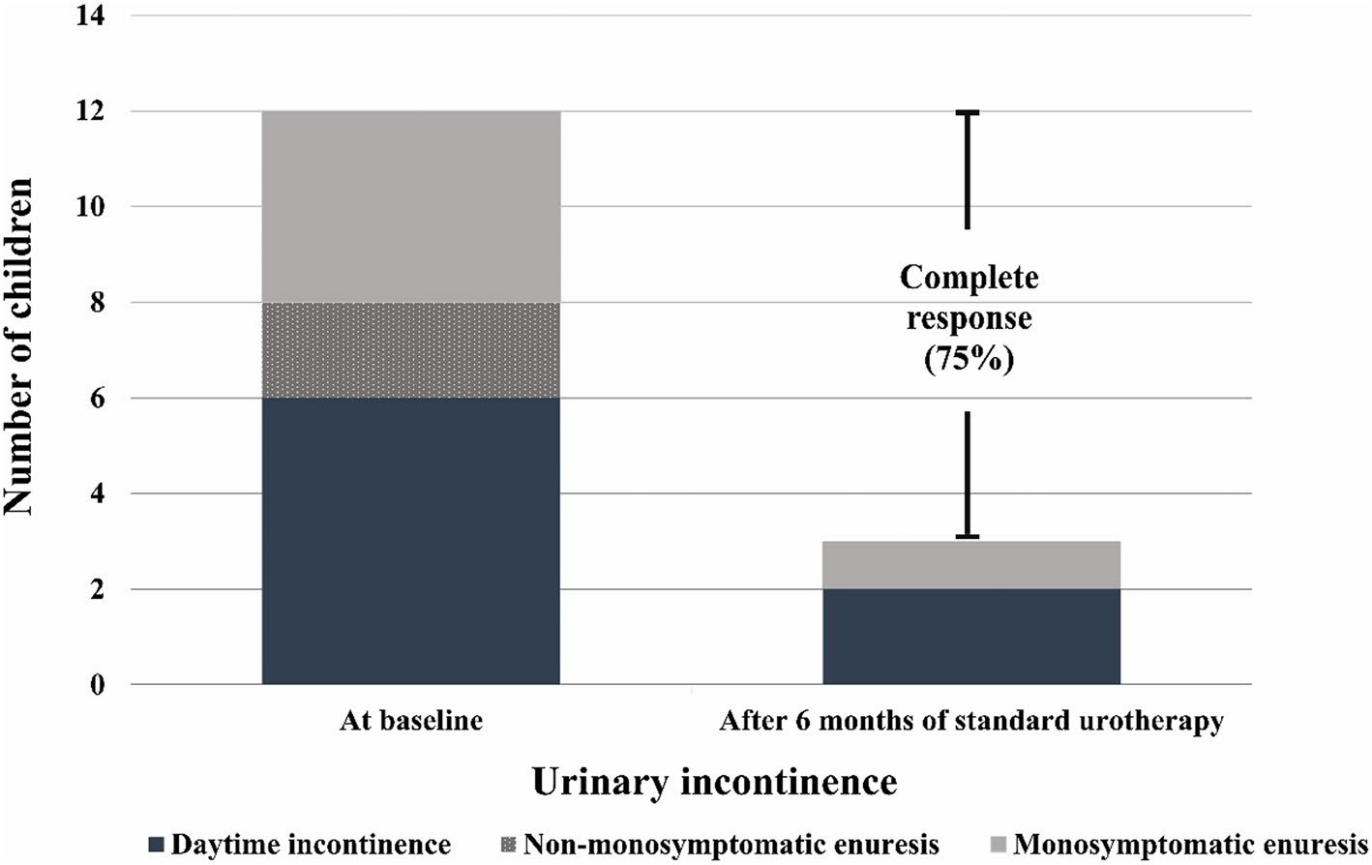
Children with urinary incontinence at baseline, and the outcomes of standard urotherapy after 6 months of treatment. Urinary incontinence included daytime incontinence (*n* = 6), non-monosymptomatic enuresis (*n* = 2), and monosymptomatic enuresis (*n* = 4). Nine of 12 children (75%) achieved urinary continence or complete response after 6 months of standard urotherapy

Among the 1,042 children who completed the DVSS questionnaire, 97 (9.3%) had urinary incontinence, and the prevalence of daytime incontinence was 7.8%. Among the 24 children who completed 6 months of standard urotherapy, 20 patients completed their bladder and bowel diaries. At baseline, 12 patients had urinary incontinence, including 6 with daytime incontinence, 2 with non-monosymptomatic enuresis, and 4 with monosymptomatic enuresis. At 6 months, 9 (75%) of those 12 children achieved urinary continence or complete response after standard urotherapy, including 4 with daytime incontinence, 2 with non-monosymptomatic enuresis, and 3 with monosymptomatic enuresis (Fig. 1). The mean overall Bristol stool score also significantly improved from 2.6 ± 0.7 to 3.2 ± 1.0 (*p* = 0.03) by the 6-month time point. One participant developed 1 episode of UTI during the follow-up period.

## Discussion

BBD is characterized by the combination of LUTD and bowel dysfunction, and can be initially diagnosed by a general practitioner or pediatrician in the outpatient department using BBD questionnaires, such as the DVSS and bladder and bowel diaries. Advanced diagnostic imaging, such as ultrasound or voiding cystourethrography, may be required – especially when patients have a history of UTI. However, in the absence of UTI risk factors, history and related questionnaires with or without uroflow are usually sufficient to make the diagnosis [1, 2, 15].

In this study, we evaluated the prevalence of BBD in children aged 4–12 years, and the treatment outcomes of standard urotherapy prescribed in a general pediatric outpatient clinic setting. Using the DVSS questionnaire, the prevalence of BBD in Thai children was 8.6%, which is lower than the rate reported among Brazilian children in a school setting by Vaz, et al. They found a prevalence of 21.8% in children aged 6–12 years, and more frequent in girls, children aged 6–8 years, and of a low socioeconomic level [7]. However, the prevalence rate found in our study is similar to the rates recently reported by Yukse, et al. and Sampaio, et al. [3, 4].

Yukse, et al. studied Turkish primary school children and reported a prevalence of LUTD of 9.3%, and more frequent in girls, in those with more siblings, in those with a past history of UTI, and in girls using the squatting position [3]. In the present study, BBD was also found to be predominant in girls, but we found no significant difference in sibling number, family income, or history of UTI between those with and without BBD. In addition, constipation is known to commonly coexist with LUTD. Sampaio, et al. conducted a population-based study and reported a prevalence of BBD of 9.1% among 829 children and adolescents with a mean age of 9.1 years. They also found that constipated children were 6.8 times more likely to have LUTD [4]. In our study, low defecation frequency and difficult defecation were the most common bowel dysfunction-related symptoms, and they were found to coexist with lower urinary tract symptoms, such as curtsying, urgency, and infrequent voiding. Daytime incontinence was found in approximately one-third of BBD in this study.

BBD was reported to be associated with psychosocial and behavioral problems [6, 16]. Duorado, et al. studied 806 children (mean age: 9.1 years) and reported an LUTD prevalence of 16.4%. Using the SDQ, they found that children with LUTD had a high rate of emotional and behavioral problems (40.5%), and BBD was found to be an aggravator [16]. In the present study, we found 17.5% of BBD children to be at risk for emotional and behavioral problems (17.5%), and that 20.0% of BBD children had emotional and behavioral problems according to overall SDQ scale scoring, and that hyperactivity was the most common problem (32.5%). It is not yet known whether BBD aggravates the behavioral issues or if the neuropsychiatric conditions precipitate BBD.

Standard urotherapy and constipation management are the mainstay of treatment for BBD, and should be given as first-line therapy [13, 17]. Pharmacotherapy of LUTD and surgical treatment should be considered only in patients who fully complied with, but failed first-line treatment after 6 months [2, 18]. In our study, we found that BBD children treated with standard urotherapy and constipation management had significant improvement in DVSS as early as 3 months, and further improvement at 6 months. Nine of 12 children with urinary incontinence demonstrated complete response to the treatment. Furthermore, the mean overall Bristol stool score was significantly improved from baseline to 6 months, which correlated with improvement in the 2 defecation-specific questions in the DVSS.

## Limitations

The main limitation of this study is that only 24 of the 41 children who agreed to participate in 6 months of standard urotherapy treatment completed the 6 months of treatment. In addition to other more common reasons for study dropout, the 2019 coronavirus pandemic occurred during our 2018–2020 study period, which may explain the higher-than-expected number of children who left the study. However and importantly, the baseline characteristics and mean total DVSS result were not significantly different when compared between those who did and did not complete 6 months of standard urotherapy treatment.

## Conclusion

BBD in children is not rare and could go unnoticed to general practitioners and caregivers. Screening for BBD using the DVSS questionnaire in a general pediatric outpatient clinic setting may be helpful for early detection and normalization of urination and bowel patterns. Education and behavior management of children and their caregivers, such as standard urotherapy, may reduce patient symptoms. In this study, standard urotherapy prescribed in a general pediatric outpatient clinic setting yielded favorable outcomes in Thai BBD children, with a 75% rate of complete response among those with urinary incontinence. Further investigation is warranted in patients that fail to sufficiently respond to 6 months of standard urotherapy. BBD is an underdiagnosed condition among children, so general practitioners and pediatricians should increase their level of awareness of this disorder, and not too hastily rule out a diagnosis of BBD.

## Data Availability

The datasets generated and/or analyzed in this study are not publicly available due to concerns about participant confidentiality, but they are available from the corresponding author upon reasonable request from the editor.

## Abbreviations

ANOVA: Analysis of variance
BBD: Bladder and bowel dysfunction
COA: Certificate of approval
DVSS: Dysfunction Voiding Symptom Score
LUTD: Lower urinary tract dysfunction
OPD: Outpatient department
Q: Question
SD: Standard deviation
SDQ: Strengths and Difficulties Questionnaire
Si: Siriraj
THB: Thai baht
UTI: Urinary tract infection
